# Differences in end-of-life health service usage between people who died at home before and during the pandemic in Scotland.

**DOI:** 10.23889/ijpds.v7i3.1880

**Published:** 2022-08-25

**Authors:** Jan Savinc, Iain Atherton

**Affiliations:** 1 Edinburgh Napier University

## Objectives

To compare health service usage of people who died at home in Scotland during the Covid-19 pandemic to the population who died at home prior to the pandemic, as a proxy measure of end-of-life care availability during the pandemic.

## Approach

Retrospective observational study of linked routine data: death registrations of all adults who died aged 18+ in Scotland during 12-month pandemic period (2020-03-23 to 2021-03-22) and pre-pandemic period (5 years prior), linked to inpatient acute hospital, psychiatric, and cancer registration records, and unscheduled care records (NHS24, A&E, ambulance, and Out-of-hours GP records). Service usage will be summarised for pandemic and pre-pandemic cohorts.

## Results

Deaths at home increased by about 35% during the first year of the pandemic in Scotland. Health service usage before and during the pandemic will be compared, in particular, length of stay in hospital in the last year of life, to test the hypothesis that patients were discharged earlier to ease pressures on hospitals. Geographical differences in length of hospital stay will be presented in terms of NHS health boards, Urban-rural status and Scottish Index of Multiple Deprivation (SIMD).

## Conclusion and Relevance

Studying the effects of the pandemic on end-of-life care has important implications for policy and future pandemic preparedness. These preliminary results are an early step towards understanding the effects of the pandemic and motivate further linkage of social care data as soon as they are made available.

